# Aggression in crime and sports: a study on prisoners and amateur combat athletes in Türkiye

**DOI:** 10.1186/s40359-024-02329-w

**Published:** 2025-01-06

**Authors:** Muhammed Sıddık Çemç, Enes Madak, Özgür Gülen, Hüseyin Ozan Sönmez

**Affiliations:** 1 Department of Physical Education and Sports, Boğaziçi University, Istanbul, 34342 Türkiye; 2 Turkish Naval Academy, National Defense University, Istanbul, 34942 Türkiye; 3 Institute of Health Sciences, Süleyman Demirel University, Isparta, 32260 Türkiye

**Keywords:** Aggression levels, Athlete psychology, Inmate psychology, Intentional injury, Rehabilitation

## Abstract

**Background:**

This research focuses on examining and comparing the aggression levels of prisoners incarcerated for intentional injury and amateur combat athletes. The study aims to explore the differences in aggression levels among these groups to understand the impact of incarceration and sports participation on aggression.

**Methods:**

The participants included in the analysis consist of prisoners (*n* = 363) housed in Marmara No. 7 and No. 3 Type L Closed Penal Institutions, who have been incarcerated for intentional injury, with an average age of 36.64 ± 10.30 years and an average custody period of 980.47 ± 1335.27 days. Amateur combat athletes (*n* = 203) with an average age of 25.17 ± 10.09 years have been actively engaged in sports for an average of 11.83 ± 9.53 years. The control group (*n* = 329) has an average age of 32.65 ± 7.56 years. Data collection instruments included a personal information form and the Buss-Perry Aggression Questionnaire. The data were analyzed using IBM SPSS Statistics 23.0, with a significance level set at *p* < 0.05.

**Results:**

Analysis of the Buss-Perry Aggression Questionnaire scores revealed that the aggression levels of prisoners incarcerated for intentional injury were statistically significantly lower than those of both amateur combat athletes and control group participants (*p* < 0.001). Additionally, a significant reduction in overall aggression scores was observed among prisoners as the number of days in custody increased (*p* = 0.045). No significant differences were found among amateur combat athletes concerning the discipline variable, and no meaningful relationship was identified between years of active sports participation and aggression scores.

**Conclusion:**

The findings indicate that educational, social, and sports activities organized in correctional facilities can be effective in reducing aggression levels among inmates. Systematic planning, professional implementation, and continuous evaluation of such programs can make significant contributions to the rehabilitation of prisoners and their successful reintegration into society.

## Introduction

The American Psychological Association [1] defines aggression as “*behavior intended to cause physical or psychological harm to another individual or group of individuals.*” Whether aggression manifests verbally or physically in interpersonal relationships or is exhibited in the form of wars or massacres, it is as old as human history. There are also socially acceptable forms of aggression in sports, economic competition, national defense, and politics. Due to the ever-changing conditions of the world, the factors that lead to aggression in individuals, the types of aggression, and the ways in which aggression is displayed are subject to variation [2].

In 1961, the World Health Organization [3] recognized violence as a major public health issue. It is observed that aggression and violence have become common behaviors in both public and private spheres in Türkiye, as in the rest of the world. According to a report published by Çuhadaroğlu et al. [4], the proportion of adolescents aged 12–21 in Türkiye who have witnessed violence at home is 17%, while the proportion who have witnessed violence at school is 34%, and those who have witnessed violence in their neighborhood is 28%.

Sports competitions provide a suitable environment for aggressive behaviors driven by competition. However, not every behavior that harms an opponent can be classified as aggression. The boundaries of aggression in sports activities are determined by the rules of the respective sport. As Tiryaki [5] notes, similar actions are evaluated differently in various sports. For example, a “neck grab” move in wrestling cannot be applied in handball. While a punch thrown at an opponent is part of a boxing match, it would be penalized in another sport. The likelihood of aggressive behavior increases with the amount of physical contact in the sport.

In most sports, athletes display aggressive behaviors by applying physical force within established rules. As a result, the degree of aggression varies from one sport to another. Certain sports, like American football, ice hockey, boxing, and wrestling, are often regarded as having a higher concentration of aggressive elements. Additionally, while it is suggested that individuals who engage in sports with a high aggression component may exhibit aggressive behaviors more frequently in their non-sporting lives, there is a counterargument that these athletes manage their anger and aggressive behaviors more effectively during their sports careers, leading to less aggressive behavior overall [6].

Research highlights that aggression is shaped by both personal traits and broader cultural influences. While individual factors such as personality and emotional regulation play a critical role, social norms and cultural values can either amplify or mitigate aggressive behaviors [7]. In this context, sports are proposed as a significant tool for managing aggression at both individual and social levels. The literature highlights that participation in sports has a mitigating effect on aggression levels [8].

The rates of incarceration are increasing both globally and in Türkiye in terms of both proportion and absolute numbers [9]. According to the 2023 prison report prepared by the University of Lausanne, as of January 31, 2023, Türkiye has the highest number of inmates among the member countries of the Council of Europe, with 348,265 individuals. Türkiye is followed by the United Kingdom (90,964), France (72,294), and Poland (71,228) [10].

In combat sports, the primary aim is to physically engage with the opponent within the framework of established rules to achieve success. The foundation of success is determined by executing regulated strikes, blows, and maneuvers against the opponent. From this perspective, social recognition and rewards often follow a victory achieved through these actions. However, when similar physical actions are applied directly and without rules in interpersonal situations, they constitute a crime, potentially leading to the arrest and conviction of the individual involved. Thus, while one scenario results in a reward, the other leads to punishment. This raises an intriguing question about how similar physical actions, depending on the context, might affect levels of aggression. This research sets out to discover and compare the aggression levels of prisoners sentenced for intentional injury and amateur combat athletes.

The research questions are as follows:

- RQ1: Is there a significant difference in aggression levels between individuals engaged in amateur combat sports and prisoners convicted of intentional injury?
- RQ2: Is there a relationship between the length of incarceration and aggression levels among prisoners convicted of intentional injury?
- RQ3: Do aggression levels of individuals engaged in amateur combat sports differ according to the type of combat sport practiced?
- RQ4: Is there a relationship between the aggression levels of individuals engaged in amateur combat sports and the duration of their sports careers?

The hypotheses of the current study are as follows:

- H1: There is no significant difference in aggression levels between individuals engaged in amateur combat sports and prisoners convicted of intentional injury.
- H2: There is no significant relationship between the length of incarceration and aggression levels among prisoners convicted of intentional injury.
- H3: The aggression levels of individuals engaged in amateur combat sports do not significantly differ based on the type of combat sport practiced.
- H4: There is no significant relationship between the aggression levels of individuals engaged in amateur combat sports and the duration of their sports careers.

## Methods

### Data collection instrument

This study utilizes a cross-sectional design. The data collection instrument is the Buss-Perry Aggression Questionnaire [11], which was adapted from the Buss-Durkee Hostility Inventory [12]. The scale was translated into Turkish, and its validity and reliability were established by Demirtaş Madran [13].

The Buss-Perry Aggression Questionnaire, one of the most frequently used aggression scales in the global literature, is a 29-item, 5-point Likert-type scale (1-Strongly disagree, 2-Disagree, 3-Undecided, 4-Agree, 5-Strongly agree). It is designed to measure four different dimensions of aggression: physical aggression, verbal aggression, anger, and hostility. The Physical Aggression subscale consists of 9 items related to physically harming others (items 2, 5, 8, 11, 13, 16, 22, 25, and 29). The Verbal Aggression subscale includes 5 items related to verbally hurting others (items 4, 6, 14, 21, and 27). The Anger subscale, aimed at measuring the emotional aspect of aggression, comprises 7 items (items 1, 9, 12, 18, 19, 23, and 28). The Hostility subscale, which targets the cognitive dimension of aggression, contains 8 items (items 3, 7, 10, 15, 17, 20, 24, and 26). Additionally, items 9 and 16 are reverse-scored.

Significant relationships have been demonstrated between each of the subscales [11, 14]. The internal consistency coefficients obtained in Buss and Perry’s [11] original study are as follows: physical aggression 0.85, verbal aggression 0.72, anger 0.83, hostility 0.77, and a total score of 0.89. In the study conducted by Demirtaş Madran [13], the Cronbach’s Alpha coefficient for the entire Buss-Perry Aggression Questionnaire [11] was found to be 0.85; the alpha value for the physical aggression subscale was 0.78; for the verbal aggression subscale, 0.48; for the anger subscale, 0.76; and for the hostility subscale, 0.71. Additionally, the test-retest reliability (Pearson correlation) coefficient for the total score was determined to be 0.97, and the correlation coefficient calculated for criterion validity was 0.49, indicating that the Turkish version of the scale is a valid and reliable measurement tool. In our study, the Cronbach’s Alpha value obtained for the entire scale was 0.893; for the physical aggression subscale, it was 0.777; for the verbal aggression subscale, 0.514; for the anger subscale, 0.776; and for the hostility subscale, 0.726.

### Data collection process from participants

This study focuses solely on male participants. The rationale for this decision lies in the significant differences in aggression mechanisms and expression patterns between genders, which could complicate the interpretation of the results. Additionally, the study excludes other sports groups to maintain its specific focus on combat sports, as these sports inherently involve controlled aggression. This approach aims to facilitate the comparison of aggression levels within regulated environments.

### Data collection process from prisoners incarcerated for intentional injury

A formal request was submitted to the General Directorate of Prisons and Detention Houses of the Ministry of Justice of the Republic of Türkiye to include detainees incarcerated for intentional injury in the study. Approval was granted on December 20, 2023, to conduct the research in the No. 3 and No. 7 Type L Closed Penal Institutions located within the Marmara Closed Penal Institution Campus in Istanbul. This campus comprises eight separate facilities, designated as Type L No. 1 through Type L No. 8. All detainees incarcerated for intentional injury are exclusively housed in the No. 3 and No. 7 facilities, allowing the study to encompass the entire population of detainees convicted for this specific crime. Importantly, the prison administration did not impose any restrictions or influence the inclusion process, ensuring an unbiased selection of participants.

Specialist personnel at Marmara No. 3 and No. 7 Type L Closed Penal Institutions ensured that the detainees participating in the study were informed and that they completed the informed consent form. Detainees and convicts incarcerated for terrorism-related offenses were not included in the study. The scales and forms used did not include the names or surnames of the detainees and convicts. Additionally, no audio or video recordings were made. Before administering the Buss-Perry Aggression Questionnaire, participants were asked to complete an information form that included questions about age, educational level, occupation, and the duration of their detention. Data were collected from a total of 385 male participants detained for the crime of intentional injury. However, 22 of these participants were excluded from data analysis due to incomplete or erroneous information.

### Data collection process for amateur combat athletes and control group participants

Initially, within the scope of the research, the informed consent form, personal background form, and Buss-Perry Aggression Questionnaire were prepared using Google Forms and were distributed to amateur combat athletes and control group participants, with a request for them to complete these forms.

Data were collected from 203 male amateur combat athletes who have not committed any crimes and continue their active sports careers, including karate (*n* = 46), taekwondo (*n* = 47), wrestling (*n* = 42), and kickboxing (*n* = 68). Before administering the Buss-Perry Aggression Questionnaire, participants were asked to complete an information form that included questions about age, educational level, occupation, sports discipline, years of sports experience, and national athlete status, as well as the informed consent form.

The study also examined a control group of 329 male individuals who had not committed any crimes and had not been involved in combat sports. Before administering the Buss-Perry Aggression Questionnaire, participants were asked to complete an information form that included questions about age, educational level, and occupation, as well as the informed consent form.

### Data analysis

IBM SPSS Statistics 23.0 software (IBM Corp., Armonk, NY, USA) was used for data analysis. Initially, the normality distribution of the obtained data was examined, and the skewness and kurtosis values were found to be between “−1.5 and + 1.5,” indicating that the data followed a normal distribution [15]. Therefore, One-Way Analysis of Variance (ANOVA) was used to determine differences in multiple comparisons, and the Bonferroni post-hoc test was employed to identify the source of the differences. Pearson Correlation Analysis was applied to determine the relationship between variables.

The skewness and kurtosis values for the normality distribution of the data are observed to be within the range of “−1.5 to + 1.5,” as shown in Table 1 [15].

**Table 1:** Normality distribution table

Scale Subdimensions	*n*	Number of Items	Mean ± SD	Skewness	Kurtosis
Physical Aggression Subdimension	895	9	20.25 ± 5.94	0.356	− 0.299
Verbal Aggression Subdimension		5	38.48 ± 2.71	− 0.206	0.620
Anger Subdimension		7	15.74 ± 5.13	0.312	− 0.501
Hostility Subdimension		8	20.24 ± 5.51	− 0.001	− 0.230
Buss-Perry Aggression Questionnaire Total		29	69.58 ± 16.27	0.088	− 0.221

*Abbreviations*: *n* Number of Participants, *SD *Standard Deviation, *Skewness *Skewness of the data distribution, *Kurtosis *Kurtosis of the data distribution

## Results

The prisoners incarcerated for intentional injury who participated in the study and were included in the analysis (*n* = 363) had an average age of 36.64±10.30 years and had been in custody for an average of 980.47±1335.27 days. The amateur combat athletes (*n* = 203) had an average age of 25.17± 10.09 years and had been actively engaged in sports for an average of 11.83±9.53 years. The control group participants (*n* = 329) had an average age of 32.65±7.56 years.

**Fig. 1:**
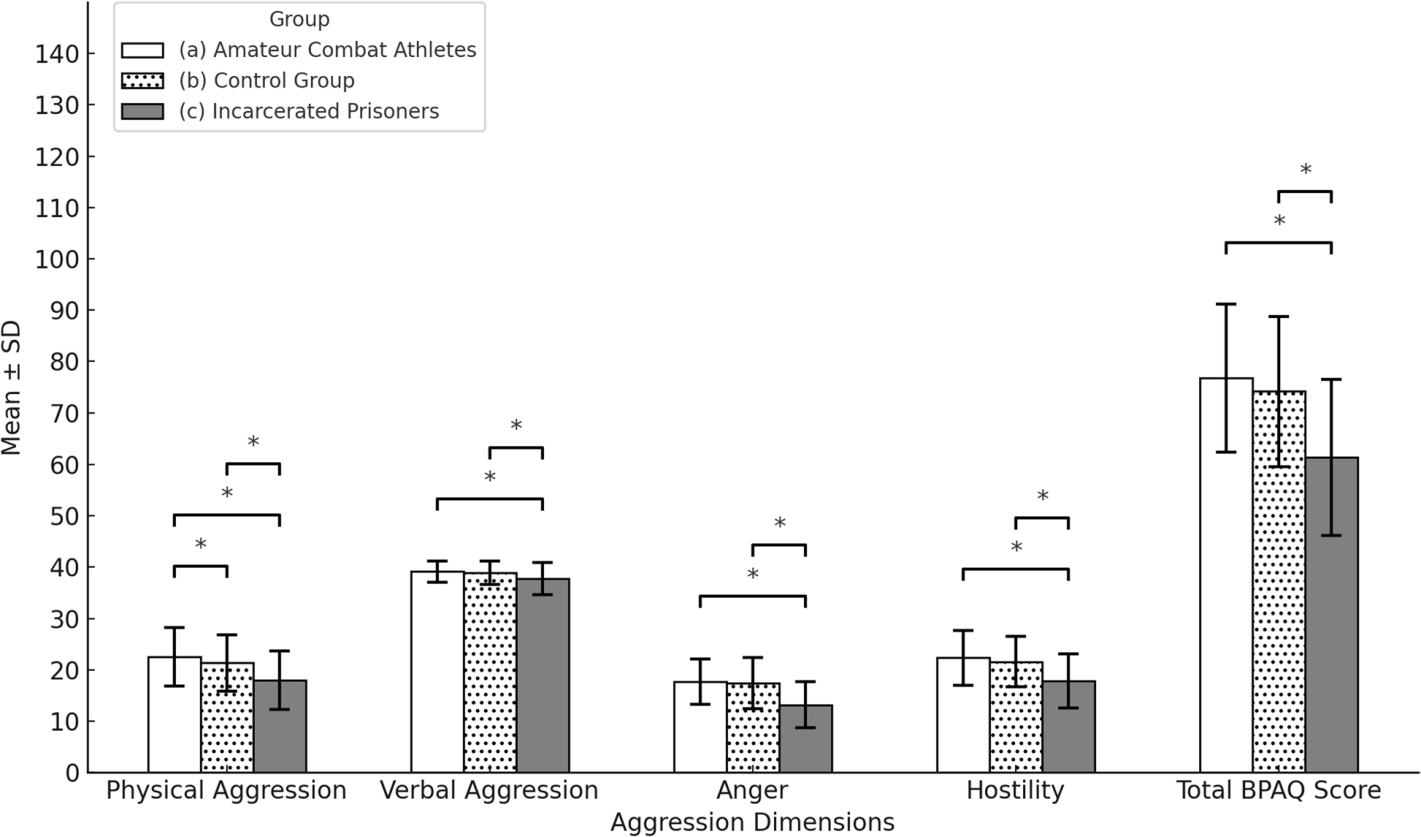
One-way ANOVA for the BPAQ and its subdimensions based on participant group variable. Abbreviations: f = F-statistic value from the ANOVA test, *p* = *p*-value indicating statistical significance (*p < 0.001 indicates statistical significance), n^2^ = Eta-squared (effect size measure). Note: Bonferroni = Bonferroni posthoc comparisons between groups (a = Amateur Combat Athletes, b = Control Group, c = Incarcerated Prisoners)

In terms of the physical aggression subscale, amateur combat athletes obtained a mean score of 22.54±5.65, the control group scored 21.32±5.54, and prisoners convicted of intentional injury scored 17.99±5.68. A statistically significant difference was observed between the groups (f = 52.283, *p* < 0.001, n^2^ = 0.105, Bonferroni = a>b, a>c, b>c). Regarding the verbal aggression subscale, amateur combat athletes scored 39.13±2.11, the control group 38.90±2.27, and prisoners convicted of intentional injury 37.73±3.16. Again, a statistically significant difference was observed between the groups (f = 25.167, *p* < 0.001, n^2^ = 0.053, Bonferroni = a>c, b>c). For the anger subscale, amateur combat athletes scored 17.71±4.43, the control group 17.38 ± 5.00, and prisoners convicted of intentional injury 13.15±4.49, indicating a meaningful statistical distinction between the two groups (f = 94.066, *p* < 0.001, n^2^ = 0.174, Bonferroni = a>c, b>c). Regarding the hostility subscale, amateur combat athletes scored 22.33 ± 5.32, the control group 21.55 ± 4.93, and prisoners convicted of intentional injury 17.88±5.26. This is also a meaningful statistical distinction between the two groups (f = 65.109, *p* < 0.001, n^2^ = 0.127, Bonferroni = a>c, b>c). When examining the total scores obtained from the Buss-Perry Aggression Questionnaire, amateur combat athletes scored 76.80 ± 14.33, the control group 74.20±14.59, and prisoners convicted of intentional injury 61.36±15.17. A statistically significant difference was observed between the groups (f = 96.492, *p* < 0.001, n^2^ = 0.178, Bonferroni = a>c, b>c). In conclusion, the scores obtained from all subscales (physical aggression, verbal aggression, anger, hostility) and the total score of the Buss-Perry Aggression Questionnaire indicate that the aggression levels of prisoners convicted of intentional injury were statistically significantly lower than those of both amateur combat athletes and individuals in the control group (Fig.1).

The analysis revealed a weak negative correlation between the number of days imprisoned and the total score of the Buss-Perry Aggression Questionnaire (*r* = −0.105, *p* = 0.045), as shown in Table 2. This indicates that as the number of days of imprisonment increases for prisoners convicted of intentional injury, there is a corresponding decrease in their level of aggression.

**Table 2:** Pearson correlation analysis of the BPAQ and its subscale scores concerning the number of days imprisoned among prisoners convicted of intentional injury

	Physical aggression subdimension	Verbal aggression subdimension	Anger Subdimension	Hostility Subdimension	Buss-Perry Aggression Questionnaire Total
r	− 0.084	− 0.073	− 0.090	− 0.081	− 0.105
p	0.109	0.163	0.087	0.124	0.045*
n	363	363	363	363	363

*Abbreviations*: *r* Pearson correlation coefficient, *p* *p*-value indicating statistical significance (*p < 0.05 indicates statistical significance), *n* Number of participants

**Fig. 2:**
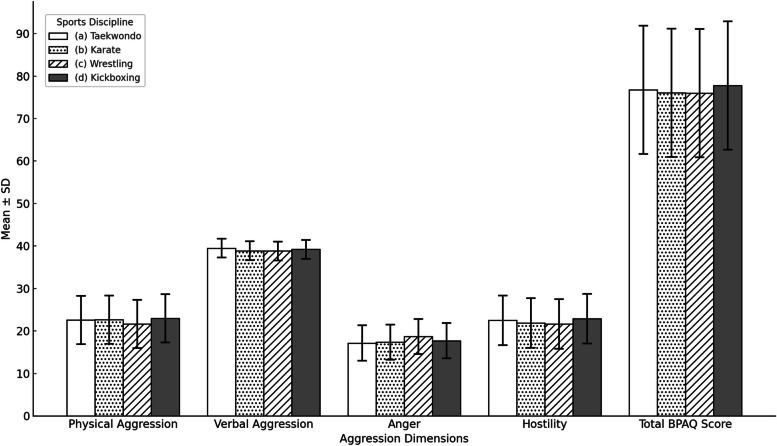
One-way ANOVA of the BPAQ and its subscales according to the sports discipline variable among amateur combat athletes. Abbreviations: f = F-statistic value from the ANOVA test, *p* = *p*-value indicating statistical significance (*p < 0.05 indicates statistical significance), n^2^ = Eta-squared (effect size measure)

In terms of the physical aggression sub-dimension, taekwondo athletes had a mean score of 22.60±5.70, karate athletes 22.67±5.30, wrestling athletes 21.64±5.99, and kickboxing athletes 22.97±5.70. No statistically significant difference was observed between the groups (f = 4.910, *p* = 0.689, n^2^= .007). For the verbal aggression sub-dimension, taekwondo athletes scored 39.49±2.22, karate athletes 38.89±1.77, wrestling athletes 38.83 ± 2.22, and kickboxing athletes 39.22±2.19 on average. Again, no statistically significant difference was observed between the groups (f = 0.965, *p* = 0.410, n^2^ = .014). In the anger sub-dimension, taekwondo athletes had a mean score of 17.17±4.14, karate athletes 17.35±4.75, wrestling athletes 18.71±4.37, and kickboxing athletes 17.71±4.43. No statistically significant difference was found between the groups (f = 1.056, *p* = 0.369, n^2^ = .016). For the hostility sub-dimension, taekwondo athletes scored 22.53 ± 5.85, karate athletes 21.91 ± 4.76, wrestling athletes 21.64±5.90, and kickboxing athletes 22.88 ± 4.96 on average. No statistically significant difference was observed between the groups (f = 0.590, p = 0.622, n^2^ = .009). When examining the total scores obtained from the Buss-Perry Aggression Questionnaire, taekwondo athletes had a mean score of 76.74 ± 15.10, karate athletes 76.07±14.07, wrestling athletes 76.02±15.25, and kickboxing athletes 77.81 ± 13.61. No statistically significant difference was detected between the groups (f = 0.192, *p* = 0.902, n^2^ = .003). In conclusion, no statistically significant differences were identified in the total scores or subscale scores of the Buss-Perry Aggression Questionnaire among amateur combat sports athletes in terms of their respective sports disciplines (Fig. 2).

Amateur combat athletes have been actively engaged in their sporting careers for an average of 11.83 ± 9.53 years, as shown in Table 3. It was observed that there is no significant correlation between the variable of years of experience in sport and the total score or subscale scores of the Buss-Perry Aggression Questionnaire.

**Table 3:** Pearson correlation analysis of the BPAQ and its subscale scores concerning the years of experience in sport among amateur combat athletes

	Physical Aggression Subdimension	Verbal Aggression Subdimension	Anger Subdimension	Hostility Subdimension	Buss-Perry Aggression Questionnaire Total
r	0.127	0.008	− 0.068	0.011	0.030
p	0.071	0.906	0.335	0.872	0.671
n	203	203	203	203	203

*Abbreviations:* *r *Pearson correlation coefficient, *p* *p*-value indicating statistical significance (*p < 0.05 indicates statistical significance), *n* Number of participants

## Discussion

Kotarska et al. [16] underscore that contact-based sports like martial arts and combat disciplines provide a distinct ability to convey moral lessons, aiding in the cultivation of positive behavior and diminishing aggression. Nosanchuk [17] compared students who were actively engaged in karate, taekwondo, and judo with those who had practiced these sports in the past but had since ceased participation. He found an inverse relationship between the students’ level of aggression and their belt rank. Further studies have demonstrated that martial arts training can lead to reductions in hostility [18], anger [19], and aggressive tendencies [20, 21]. Participation in these exercises has also been shown to promote greater relaxation and empathy [22], increase self-confidence [23] and self-esteem [24], and enhance self-control skills [19].

Graczyk et al. [25] reported that engaging in sports, particularly martial arts (karate, taekwondo, aikido), has a constructive impact on socialization. Lamarre and Nosanchuk [26] claimed that judo training reduces aggression based on their research findings. Additionally, Szabo and Parkin [27] and Boostani et al. [28] found that martial artists exhibit less aggression compared to non-athletes. Daniluk et al. [29] noted that members of the national judo team displayed low to moderate levels of aggression. Budnik [30] found out that the karate group showed decreased aggression levels compared to a control group consisting of students. Ziaee et al. [31] indicated that karate instruction has productive effects on managing aggression in pre-adults. Kostorz and Sas-Nowosielski [32] examined the aspects of aggression in sportsmen engaged in martial arts and combat sports. Their findings revealed that martial artists exhibit statistically significantly lower levels of aggression compared to combat sports athletes. Research by Reynes and Lorant [33] indicated that non-athletes exhibit higher levels of aggression compared to athletes engaged in martial arts and combat sports. Pačesová and Šmela [34] examined aggression levels among non-athletes, athletes participating in contact sports, and those engaged in non-contact sports. Their study included 47 contact sport athletes, 51 non-contact sport athletes, and a control group of 55 individuals. The results showed that non-athletes were physically more aggressive and demonstrated higher overall levels of aggression compared to contact sport athletes. Moreover, non-athletes were observed to be both physically and verbally more aggressive than non-contact sport athletes.

Basiaga-Pasternak and Ambrozy [35] found that professional fighters, specifically in kickboxing and MMA, exhibit lower levels of aggression. Nonetheless, they found no significant statistical distinction in aggression levels between elite and non-professional fighters. They suggested that engaging in combat sports over many years while adhering to rules could be effective in reducing aggression levels among athletes. Kubacka-Jasiecka and Wrzesniewski [36] demonstrated that individuals who have undergone long-term karate training exhibit lower levels of aggression compared to novices. Chen et al. [37] also identified a decrease in aggression associated with prolonged experience in combat sports. Kurpel et al. [38] reported in their study that increased competition experience among wrestlers is associated with a reduction in aggression. Similarly, Druminska et al. [39] observed a decline in overall aggression with increased experience among kids who are 12–15 and practice judo, taekwondo, and karate.

Kuśnierz et al. [40] examined aggression levels among individuals practicing combat sports and martial arts compared to a control group. The study included 150 men practicing capoeira, boxing, and jiujitsu (50 participants from each discipline) and a control group of 150 secondary school and university students. The results indicated that the highest level of aggression was recorded in the boxing group, while the lowest level of aggression was observed among jiujitsu practitioners. Notably, the control group exhibited the highest level of general aggression. The researchers concluded that training in combat sports and martial arts might provide an opportunity to diffuse emotions and reduce tension, thereby potentially lowering aggression levels. Mroczkowska et al. [41] found that wrestlers demonstrated higher levels of aggression compared to karate practitioners. Vertonghen et al. [42] reported that kickboxers and Muay Thai practitioners displayed higher levels of physical aggression than those practicing judo, aikido, and karate.

Contrary to the findings of earlier studies, Barczak et al. [43] examined the aggression levels of 198 combat athletes and a control group of 357 individuals, revealing significantly higher levels of physical aggression and overall aggression among combat athletes. Similarly, Malinauskas et al. [44] identified combat athletes as the most anger-prone group in their study. Szmajke and Dolinski [45] observed that young athletes engaged in “high-contact” sports demonstrated a greater propensity for aggressive behaviors, both in sports and daily life, compared to those without such experience. Wrzesniewski [46] found that taekwondo athletes exhibited elevated amounts of aggression across all aspects in contrast to a reference group made up of students studying sports science and rehabilitation. Basumatary [47] compared aggression levels between athletes involved in combat sports and those in non-combat sports, finding that combat athletes exhibited significantly higher levels of aggression, likely related to the intense competition inherent in these sports. Boostani and Boostani [48] reported that kickboxers had higher levels of anger, physical aggression, and hostility compared to swimmers, karate practitioners, and non-athletes, with no significant differences observed between the latter groups. Mazur and Organista [49] found that women involved in kickboxing and wrestling exhibited higher physical aggression compared to those in artistic gymnastics and synchronized swimming. Additionally, female wrestlers displayed significantly higher levels of suspicion and guilt compared to female kickboxers. Research by Chahal and Chaudhary [50], Rathod and Pujari [51], and Sofia and Cruz [52] consistently showed that contact sports athletes were more aggressive than those in non-contact sports. Zalech [53] reported a significant link between the length of training and all types of aggression, excluding bodily aggression, among taekwondo practitioners. Rotter et al. [54] raised concerns by highlighting that youths who engaged in regular sports activities 3–4 times a week exhibited significantly higher levels of bodily aggression compared to their less active peers.

Ronke and Happiness [55] conducted a study in Makurdi, Nigeria, comparing the aggression levels of prisoners with those of non-incarcerated individuals. Their findings revealed that prisoners exhibited significantly higher levels of both physical and verbal aggression compared to non-prisoners. In a parallel study conducted in 11 prisons across the United Kingdom, a significant difference in physical aggression was also observed between prisoners and non-prisoners [56]. Delisi et al. [57] examined the physical aggression levels among 831 male prisoners in the southwestern United States, focusing on those involved in street gangs, prison gangs, and both types of gangs. The study found significant differences in physical aggression behaviors between chronic offenders and non-offenders. Frigout et al. [58] aimed to evaluate aggressive behaviors by comparing prisoners and club karate athletes during karate training. Over a 26-month period in France, data were collected from 75 prisoners (55 men and 20 women) and 117 club athletes (80 men and 37 women). The results showed that both male and female club athletes exhibited significantly higher levels of aggression compared to prisoners.

Our research findings indicate that amateur combat athletes and individuals in the control group exhibit significantly higher levels of aggression compared to prisoners incarcerated for intentional injury, as evidenced by the total scores and all subscales of the Buss-Perry Aggression Questionnaire, including physical aggression, verbal aggression, anger, and hostility (Fig. 1). Furthermore, a weak negative correlation was identified between the length of incarceration and the total aggression scores among prisoners (Table 2), suggesting that longer periods of imprisonment might be associated with a reduction in aggression levels. In Türkiye, correctional institutions prioritize educational and rehabilitative activities to enable inmates to reintegrate into society as responsible individuals, prevent recidivism, and continue their lives with a sense of responsibility. These institutions offer educational opportunities ranging from basic literacy to university education and emphasize the importance of participation in specific educational and rehabilitation programs prior to release. The current Turkish correctional system functions as an educational, rehabilitative, and social reintegration center, adhering to international standards. Inmates are provided with opportunities to improve literacy, pursue education at various levels, engage in preparatory courses and library activities, participate in sports such as football, basketball, volleyball, badminton, and table tennis, acquire vocational skills in prison workshops, engage in handicraft activities, and participate in cultural and social events. A detailed examination of these social, cultural, and sporting activities underscores the comprehensive approach to inmate rehabilitation implemented by Türkiye’s correctional institutions [59].

To lessen the physical and psychological negative effects of incarceration on inmates, a variety of social and cultural activities are organized within correctional institutions, including theater, music, painting, folk dance, handicrafts, sports competitions, chess, language courses, conferences, and concerts. Inmates have the opportunity to participate in these activities either as spectators or as active contributors. Additionally, they have access to mass communication tools that are available in sufficient quantities in every correctional facility. Sporting activities, which are given special emphasis, are organized based on the physical conditions of the institution and may include team sports such as volleyball, basketball, and football, as well as individual activities like table tennis, weightlifting, and badminton. Under a protocol with the Ministry of Youth and Sports, various sports activities are organized, and training is provided by appointed referees and coaches to develop the institution’s staff in this area. The necessary equipment for sports activities in correctional facilities is supplied by the Provincial Directorates of Youth and Sports under this protocol, and various projects are also implemented within this framework. Additionally, conferences or seminars are held at least once a month in cooperation with various organizations, and these events take place in the institution’s conference and theater halls or other suitable educational environments. In open prisons, inmates are also allowed to participate in sports competitions outside the institution with various organizations, thereby fostering stronger social communication and interaction.

The findings of this study have rejected the hypotheses regarding the difference in aggression levels between prisoners convicted of intentional injury and individuals engaged in amateur combat sports (H1), as well as the relationship between the duration of incarceration and aggression levels (H2). However, the hypotheses concerning the impact of the type of combat sport (H3) and the duration of the sports career (H4) on aggression levels could not be rejected and warrant further investigation.

Our research results support the potential impact of prison policies in Türkiye on reducing aggression levels. The educational and rehabilitation programs implemented in correctional facilities aim to enhance individuals’ social adaptation and reintegration into society. Sports contribute significantly as a key component of these programs. In this context, the observed decrease in aggression levels among prisoners in our study suggests that such programs may be effective. However, the relatively low aggression levels observed among prisoners may also be attributed to social desirability bias within the prison environment. The self-report scale used in the study may have allowed participants to manipulate their responses in order to present themselves in a socially desirable manner. In environments where social norms are strong, prisoners may be inclined to report lower aggression levels than they truly exhibit. Research based on self-report scales, in particular, is vulnerable to the influence of such biases. A review of the literature indicates that social desirability bias, defined as the tendency of individuals to present themselves favorably to others, poses a significant challenge to the reliability of self-reported data. This issue is especially pronounced among populations such as sexual offenders, individuals involved in violent robberies, and those with substance abuse problems [60]. Therefore, the findings of our study require a combined consideration of both the positive effects of rehabilitation programs and the potential role of social desirability bias.

### Limitations and future research recommendations

The research was conducted at Marmara 3 and 7 No. L-Type High-Security Prisons in Istanbul, focusing on male prisoners incarcerated for intentional injury. As such, the findings may not be generalizable to other gender groups, and results may vary across different regions and countries.

The self-report scale used in the study may have allowed participants to manipulate their responses in order to present themselves in a socially desirable manner. Additionally, as of January 31, 2023, Türkiye holds the highest number of inmates among Council of Europe member countries, with 348,265 incarcerated individuals. In this context, the factors of social desirability bias and prison overcrowding must be considered, as they may impact the accuracy of self-reported measures. Such conditions may lead to underreporting of aggression levels by prisoners. The use of observation techniques and different data collection methods in future research would be beneficial in enhancing the reliability of the findings. Furthermore, the study was conducted within a specific time frame, meaning that fluctuations in aggression levels over time were not considered. Factors such as the type, frequency, and participation rates in educational, social, and sporting activities conducted within the prisons were also not taken into account. Consequently, more comprehensive studies are required to fully understand the impact of these variables.

Moreover, the environment in which individuals are situated plays a crucial role in personality formation and can significantly impact aggression levels. In the context of rehabilitation, understanding how combat sports may contribute to personality development and behavior modification is essential. The role of the environment in shaping the personality of individuals involved in combat sports, including factors such as social interactions and the nature of the sporting activities, warrants further investigation. Research has shown that success in combat sports can influence perceptions of self and success, which may have implications for aggression management and rehabilitation [61]. Additionally, studies on the personality profiles of combat sports champions versus other individuals suggest that the context in which one trains and competes can significantly shape one’s behavior and aggression [62].

It is recommended that the continuation and expansion of educational and rehabilitation programs within these institutions be prioritized and that further research be conducted to more thoroughly examine the impact of combat sports on individuals’ aggression levels, as well as their role in personality development and rehabilitation. Additionally, future research should focus on understanding the unique challenges and dynamics of conducting studies within prison settings. This includes investigating the influence of environmental factors such as overcrowding, the availability and quality of rehabilitative programs, and the potential impact of social desirability bias on self-reported data. Developing methodologies that minimize these biases, such as incorporating indirect questioning techniques or combining self-reports with behavioral assessments, can significantly improve the validity and reliability of findings in studies involving incarcerated populations. Such research would provide a more nuanced understanding of the role of institutional environments in shaping behavior and inform the development of more effective interventions.

## Conclusion

The social, cultural, and sporting activities conducted for incarcerated individuals aim to provide positive contributions to their well-being. The activities implemented align with the findings of our research, which suggests that aggressive behaviors in prisons might be balanced through various physical activities [63]. Hostile and antisocial behaviors might be reduced via enhanced self-management therapies like participation in sports [64]. The promotion of sports within prisons can assist inmates in coping with anxiety and stress [65–67], preventing depression [68], adapting and improving their behavior to reduce violations of norms and rules [65, 69–71], and reducing aggression within the prison [67, 72]. Engaging in physical activity within prisons is an effective way to improve prisoner well-being and promote social rehabilitation [73, 74]. However, achieving these goals requires appropriate methods and educational programs, as sports should not be practiced solely for physical activity [75]. In this regard, individual and team sports programs should be diversified to address the physical and psychological needs of prisoners. It is believed that conducting these activities under the supervision of professional trainers and psychologists would be beneficial for controlling aggression. Furthermore, the effectiveness of these sports programs can be enhanced by systematically examining the changes they produce in prisoners and incorporating feedback obtained from regular evaluations. In conclusion, educational, social, and sports activities implemented in correctional institutions play a significant role in reducing aggression levels among inmates. The systematic planning, professional execution, and continuous assessment of such programs can make substantial contributions to the rehabilitation of prisoners and their reintegration into society.

## Data Availability

The datasets used and/or analyzed during the current study are available from the corresponding author on reasonable request.
